# Effectiveness of Inner Traction-Facilitated Endoscopic Submucosal Dissection Using Rubber Band and Clips for Colorectal Neoplasms: A Propensity Score-Matched Study

**DOI:** 10.5152/tjg.2023.22446

**Published:** 2023-04-01

**Authors:** Guanyi Liu, Xinyue Guo, Yunlong Cai, Long Rong, Weidong Nian, Jixin Zhang

**Affiliations:** 1 Endoscopy Center, Peking University First Hospital, Beijing, China; 2 Department of Pathology, Peking University First Hospital, Beijing, China

**Keywords:** Clip and rubber band, colorectal neoplasm, endoscopic submucosal dissection, resection speed, traction

## Abstract

**Background:**

Colorectal endoscopic submucosal dissection is a technically demanding but effective treatment for superficial neoplasms. We conducted a study to compare the effectiveness and safety of inner traction-facilitated endoscopic submucosal dissection using rubber band and clip with conventional endoscopic submucosal dissection.

**Methods:**

We retrospectively evaluated 622 consecutive patients who underwent colorectal endoscopic submucosal dissection between January 2016 and December 2019. To overcome selection bias, we used propensity score matching (1:4) between endoscopic submucosal dissection using rubber band and clip and conventional endoscopic submucosal dissection. The frequency of en bloc resections, R0 resections, curative resections, procedure speed, and complications were evaluated.

**Results:**

After propensity score matching, 35 patients were included in the endoscopic submucosal dissection using rubber band and clip group and 140 were included in the conventional endoscopic submucosal dissection group. Endoscopic submucosal dissection using rubber band and clip resulted in a significant increase in resection speed (0.14 vs. 0.09 cm^2^/min; *P* = .003). There were no significant differences in en bloc, R0, and curative resection rates between the 2 groups. In subgroup analysis, the resection speed of endoscopic submucosal dissection using rubber band and clip was significantly higher than that of conventional endoscopic submucosal dissection when the lesions were equal to or larger than 2 cm, macroscopically presenting as lateral spreading tumor, and located in transverse colon to ascending colon.

**Conclusions:**

Endoscopic submucosal dissection using rubber band and clip is safe and effective in treating colorectal neoplasms, especially in lesions presenting a particular difficulty.

**Main Points**

- Endoscopic submucosal dissection using rubber band and clip (RAC-ESD) using only rubber band from gloves and clips was a safe, effective, and cheap method to treat colorectal neoplasms.
- Endoscopist in countries without particular endoscopic submucosal dissection (ESD) traction appliances can easily make rubber-band traction loops out of medical examination gloves.
- Endoscopists may actively practice RAC-ESD especially in patients with larger lesions, lesions presenting as lateral spreading tumor, and lesions located in the right colon to achieve a safe and effective ESD.

## INTRODUCTION

Endoscopic submucosal dissection (ESD) has become the standard treatment for superficial neoplasms with low risk of lymph node metastasis throughout the gastrointestinal tract.^1^ Compared with ESD in the esophagus and stomach, ESD in the colon is more technically demanding and risky due to narrow lumen, bowel movements, thinness of the colonic muscularis propria, and the poor scope maneuverability. Consequently, the complication rate of colorectal ESD has been reported to be relatively high, especially the perforation rate.^2,3^

Good and constant exposure of the submucosal space is critical for safe and quick ESD. Several traction techniques have been developed worldwide to make ESD a more effective and safer technique.^4^ The external grasping forceps-facilitated ESD is limited by the lesion location.^5^ The traction direction of dental floss-traction method cannot be adjusted freely when the lesion located deep in the colon and is assistant demanding.^6^ Magnetic anchor and balloon-assisted methods require special devices.^7,8^

In this study, a modified ESD named inner traction-facilitated ESD using rubber band and clip (RAC-ESD) was designed and evaluated for colorectal neoplasm. This study aimed to evaluate the effectiveness and safety of RAC-ESD in colorectal lesions compared to conventional ESD.

## MATERIALS AND METHODS

### Patients

This study was a single-center, retrospective, cohort study comparing the effectiveness and safety of RAC-ESD and conventional ESD in patients with colorectal neoplasms. This study was approved by the medical ethics commitee of Peking University First Hospital. All patients provided written informed consent before the endoscopic procedure.

**Figure 1.**
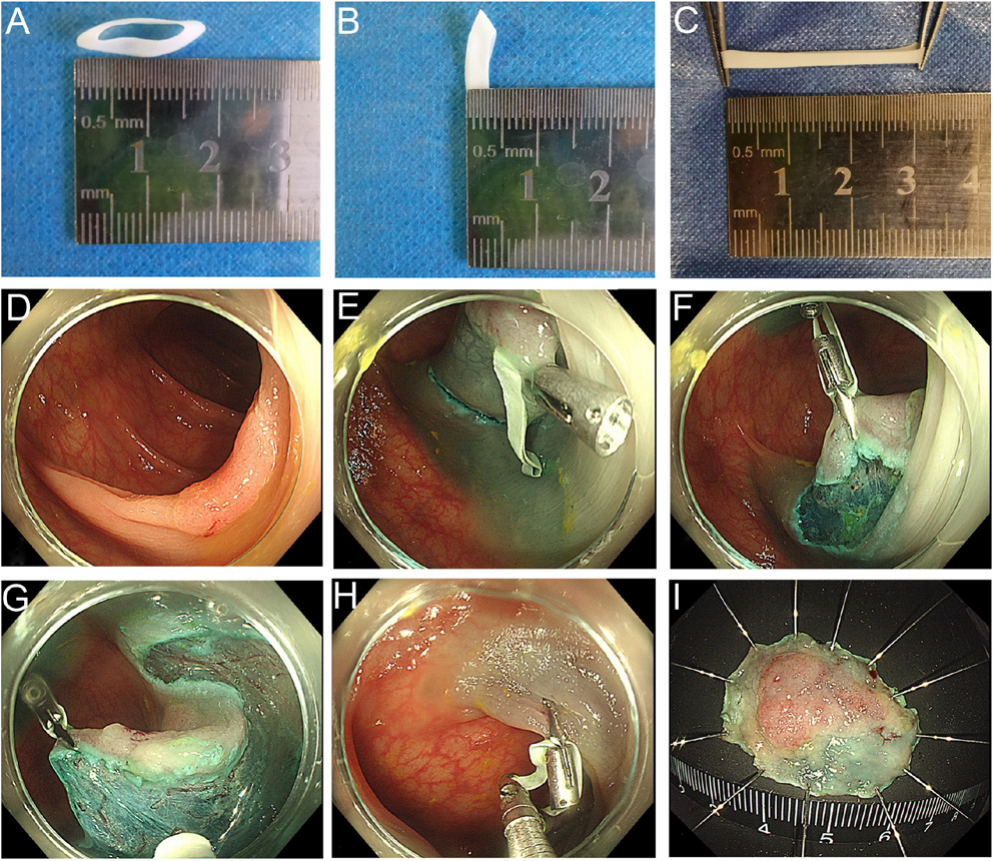
A case of inner traction-facilitated endoscopic submucosal dissection (ESD) using rubber band and clip (RAC-ESD). (A, B) Rubber bands made of latex glove were prepared before ESD, usually with a semi-parameter of 15-20 mm and a width of 2-3 mm. (C) The elasticity of the rubber band makes it more adaptable in the ESD practice. (D) A lesion of lateral spreading tumor - nongranular- flat (LST-NG-F) type was found located in the transverse colon. (E) After making a circumferential dissection, the first clip holding the rubber band was fixed at the distal part of the lesion. (F) The second clip fixed the rubber band to the contralateral colonic wall, fully exposing the submucosal layer. (G) Dissection could be safely and effectively performed. (H) The specimen was removed with cutting forceps. (I) The specimen was clear and intact.

Between January 2016 and December 2019, a total of 622 consecutive patients underwent colorectal ESD in Peking Univeristy First Hospital. After excluding patients with missing data including endoscopic or pathological results and patients treated with modified ESD such as ESD with snare, 563 ESDs were included in the study (Figure 1).

### Procedure

Endoscopic submucosal dissections were performed under general anesthesia or without accompanying sedation according to the patients’ basic condition. Patients taking anticoagulants or antiplatelet drugs consulted cardiologists to decide whether they could change to heparin or discontinue the drugs 5-7 days before ESD.

All ESDs were performed using therapeutic endoscope (GIF-Q260J or PCF-Q260JI, Olympus, Tokyo, Japan) and a transparent hood (D-201-11304, Olympus). A 1.5-mm Dualknife, IT-nano knife (Olympus), and endoclips (HXROCC-D-26-195-C, MICRO-TECH, Nanjing, China) were used. All ESDs were performed by Dr. W. N. and Dr. L.R., who were both highly experienced in ESD operation. Whether RAC-ESD was used in the operation or not was according to the operators’ decision.

**Figure 2.**
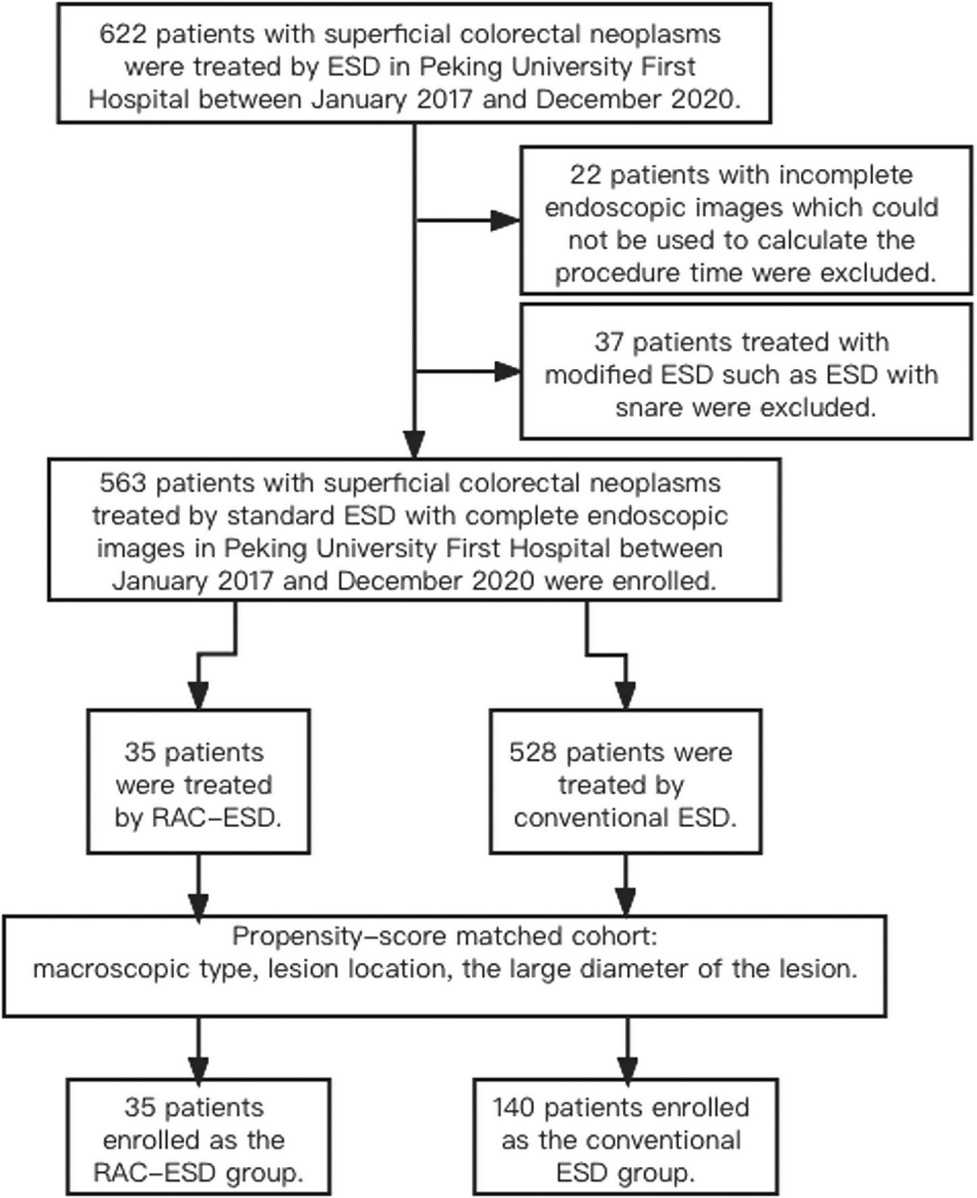
Study flow diagram. ESD, endoscopic submucosal dissection; RAC-ESD, inner traction-facilitated ESD using rubber band and clip.

In the RAC-ESD group, after making a hemicircumferential or circumferential mucosal incision and some submucosal dissection, a rubber band of 2-3 mm width and 15-20 mm semi-parameter was prepared from the medical latex glove and placed through the operative channel with a clip. The rubber band was fixed at the distal part of the lesion and then affixed with another clip to the contralateral colonic wall to expose the submucosal space (Figure 2). The strength of traction can be adjusted with the insufflation and deflation of CO_2_. When the primary traction was not sufficient enough to expose the submucosal space, or in large lesions involving different directions of the colon wall, a third or fourth clip could be applied to obtain constant exposure of submucosal layer and to change the traction direction.

### Statistical Analysis

Specimen size, procedure time, dissection speed, complications, and pathological result were recorded. An en bloc resection was defined as the procedure in which the lesion was resected in a single piece. An R0 resection was defined as the procedure in which the lesion was resected with tumor-free vertical and lateral margins confirmed pathologically. A curative resection was defined as an R0 resection without risk of lymph node metastasis, such as lymphovascular invasion, poorly differentiated components, presence of budding, or submucosal infiltration deeper than submucosal 1 (SM1, submucosal invasion more than 1000 μm). The procedure time (minutes) was defined as the time between injection and removal of the lesion. Specimen size was calculated by the ellipse formula: large diameter/2 × small diameter/2 × π (cm^2^). Dissection speed was defined by the specimen size divided by procedure time (cm^2^/min). Complications included perforation defined as a hole through the colonic wall during the procedure or clinical evidence of free gas upon postoperative abdominal tomography and bleeding defined as bleeding after ESD requiring endoscopic, interventional, or surgical treatment.

Propensity scoring was used to address the imbalance of potential confounders that may interfere with the results and was calculated using macroscopic type [lateral spreading tumor (LST) or not], location, and the large diameter of the lesion. We chose 1:4 matching using nearest neighbor matching with a caliper of width 0.1. Only patients matched with propensity scores were included in the statistical analysis. The statistics of L1 measure after matching is 0.150, far less than 0.496 before matching, indicating a promising matching result.

Matched data are presented as frequencies and percentages for categorical data or a median and interquartile range or mean and SD for continuous data. The Fisher’s exact test and the χ^2^ test were used for comparisons involving categorical variables and the Wilcoxon rank-sum test and the Student’s *t*-test were used for comparisons involving continuous variables. All *P* values were 2-tailed, and *P* < .05 was considered significant. All statistical analyses were performed using Statistical Package for Social Sciences version 27.0 (IBM Corp., Armonk, NY, USA).

## RESULTS

In total, 563 patients were eligible for inclusion. Of these, 35 patients received RAC-ESD, and 528 underwent conventional ESD. The RAC-ESD group and the conventional ESD group differed with respect to the macroscopic type, location, and the large diameter of the lesion. In order to mitigate the effects of these confounders, patients were matched at a 1:4 ratio, resulting in 35 in the RAC-ESD group and 140 in the conventional ESD group.

### Baseline Characteristics

The baseline characteristics of patients in both groups are shown in Table 1. There were no differences in the demographic characteristics, characteristics of the lesion, and pathological results.

### Outcomes

There were no significant differences in the resected area (cm^2^), with 6.0 (2.5-9.9) versus 4.1 (2.8-6.9) for the RAC-ESD and the conventional ESD group (*P* = .233), respectively. However, there were significant differences in procedure time [40.0 (25.0-80.0) vs. 30.0 (16.3-55.0) minutes; *P* = .031] and resection speed [0.14 (0.07-0.27) vs. 0.09 (0.04-0.16) cm^2^/min; *P* = .003] of the RAC-ESD and conventional ESD groups, respectively.

En bloc resection was achieved in 100.0% (35/35) of cases in the RAC-ESD group and 94.3% (132/140) of cases in the conventional ESD group. R0 resection was achieved in 97.1% (34/35) of cases in the RAC-ESD group and 95.0% (133/140) of cases in the conventional ESD group. Curative resection was achieved in 94.3% (33/35) of cases in the RAC-ESD group and 84.3% (118/140) of cases in the conventional ESD group. There were no significant differences in all these resection rates between 2 groups.

Among the 24 patients without curative resection, 8 had non-assessable horizontal margins due to piecemeal resection, 5 had positive horizontal margins, and 11 had submucosal deep-invasive cancer >1000 μm. Patients with non-assessable or positive horizontal margins were intimately followed up endoscopically. Seven of the 11 patients with submucosal deep-invasive cancers underwent additional surgery and 4 decided to receive endoscopic and radiological follow-up.

In the subgroup analyses, the differences of resection speed were significantly associated with tumor location, macroscopic type, and tumor size (whether diameter ≥ 2cm or not). For lesions located in the transverse colon to ascending colon, the resection speed was significantly higher in the RAC-ESD group than that in the conventional ESD group (0.08 vs. 0.14, *P* = .001). Compared with non-LST lesions, the resection speed for LST lesions was higher in the RAC-ESD group than that in the conventional ESD group (0.08 vs. 0.14, *P* = .001). For lesions with diameter equal to or larger than 2 cm, the resection speed was also significantly higher in the RAC-ESD group than that in the conventional ESD group (0.10 vs. 0.14, *P* = .002).

Perforation occurred in 2 cases (1.4%) in the conventional ESD group, 1 managed by endoscopic closure and the other required surgery for his elder age and the development of peritonitis. No perforation occurred in the RAC-ESD group. One patient (0.7%) in the conventional ESD group experienced delayed bleeding 1 day after procedure and was managed by electric coagulation hemostasis and clips. No other adverse events related to traction procedure occurred in the study.

After 24 (6-60) months of follow-up, 2 patients (1.4%) of the conventional ESD group developed local recurrence at 1-year follow-up and were both managed by repeated ESD, 1 of which was with positive horizontal margin. No occurrence was found in the RAC-ESD group. One patient died of ruptured abdominal aortic aneurysm 46 months after ESD.

## DISCUSSION

Oyama^9^ published the first study about countertraction in ESD in 2002, using the clip with line method. After that, endoscopists around the world worked on different traction strategies to facilitate ESD procedure. Xia et al^10^ reported in a meta-analysis that traction ESD was superior to standard ESD in terms of perforation rate and resection speed, confirming the importance of traction method.

In our study, the procedure time of the RAC-ESD group was longer than that of the conventional ESD group. We believe that this can be explained by the fact that the resected area of the RAC-ESD group was slightly larger than that of the conventional ESD group, although the difference was not significant. Overall, the resection speed of the RAC-ESD group was significantly faster than that of the conventional ESD group. The en bloc resection rate, R0 resection rate, and the curative resection rate of both groups are quite satisfactory, similar to the other studies in Asia.^10,11^ There was no significant difference in oncological and safety data between the 2 groups, which may be owing to the fact that operators involved in this study were experienced in the ESD technique. The learning curve in a previous study confirmed that ESD with traction could also be used in the training progress of inexperienced ESD operators,^12,13^ as it continuously optimizes the exposure of the submucosal space, making the process of submucosal dissection much easier.

Several studies have demonstrated the effectiveness of inner traction method in colorectal ESDs using clips with either rubber band or looped thread.^12,14,15^ In their study, the rubber band was already a commercial product but has not been approved for medical use in many other countries. And the process of making looped thread was comparatively complicated, since they need to measure and cut the thread into an appropriate length and do the knot-tying process. In our study, we use a kind of rubber band made of latex examination gloves, which are extremely easy to get in medical centers, suitable to be cut into circles, and above all inexpensive. Unlike several other previously reported traction systems, the rubber band and clip can pass through the working channel of the scope without scope removal. The elasticity of the rubber band makes it more adaptable in the ESD practice. Operators can increase or decrease the countertraction by controlling the air volume in the colorectal space. Moreover, when dealing with the giant lesions which cannot be handled by a single traction, a third or a fourth clip can be applied for traction, attaching the rubber band to the opposite site of the remnant lesion to obtain continuous countertraction as we have previously reported.^16^

Although many previous studies have confirmed the superiority of traction ESD compared with conventional ESD, still there were few studies discussed under what circumstances could traction method show the greatest effect. The subgroup analysis of our study showed that RAC-ESD improved the resection speed in lesions located in the transverse colon to ascending colon, lesions macroscopically presenting as LST, and lesions with large diameter equal to or greater than 2 cm. Endoscopic submucosal dissection is not easy to perform in transverse colon to ascending colon owing to the poor scope maneuverability and limited use of gravitation. Iacopini et al^17^ reported that positional changes did not harness the power of gravity in 22% of colonic ESD cases compared with only 3% of rectal ESD cases. Moreover, lesions presenting as LST and larger lesions are also thought to be more challenging for the endoscopists, especially inexperienced endoscopists, to perform ESD, as the technical difficulty of the procedure increases with the size of the lesion. Thus, the advantage and high efficiency of RAC-ESD over traditional ESD for lesions located in transverse colon to ascending colon, larger than 2cm or presenting as LST were more obvious. And RAC-ESD is superior in these lesions even for skillful endoscopists, as the operators included in our study are both highly experienced in ESD (performing more than 500 colorectal ESD).

The propensity score-matched method was used to reduce bias in this study. Propensity scoring was calculated by using the abovementioned confounders, including localization, macroscopic type, and the large diameter equal to or larger than 2 cm or not, as these confounders should possibly influence the difficulty of the ESD process and thus interfere with the clinical outcomes between groups. These basic characteristics were balanced between groups after propensity score matching, making the technical data between the RAC-ESD group and the conventional ESD group more comparable.

There are a few limitations to this study. First, it was a single-center, retrospective study susceptible to selection bias. Although the use of propensity score matching allowed us to balance the 2 groups, some confounders that were not incorporated into the propensity score may still influence the results. However, we consider it difficult to conduct randomized trials between RAC-ESD and traditional ESD as the former has shown to be much superior in challenging cases. Second, the 2 operators included in our study are both experienced endoscopists in ESD. We did not discuss the effectiveness of RAC-ESD strategy in the learning process for trainees in ESD. Third, we did not take the factors such as gravity, fibrosis, or difficult locations into consideration as the record of these factors could not be fully accessed, owing to the retrospective nature of the study. However, during ESD operation, operators actually tended to choose traction method in challenging cases, such as lesions with severe fibrosis or lesions located between the folds or where gravity cannot be used. Under such circumstances, RAC-ESD still showed priority to conventional ESD. Thus, this study may still underestimate the efficacy of traction. Finally, as the rubber band we used in this study was made out of latex gloves, although we did not encounter any allergy complications during the traction process, we are unaware that whether patients being allergic to latex would develop an allergic reaction when the latex come into contact with the colonic wall. Therefore, for patients with a definite medical history of severe latex allergy, RAC-ESD should be avoided in the process of endoscopic treatment.

In conclusion, RAC-ESD is a safe, effective, and cheap method to treat colorectal neoplasms, especially in lesions presenting particular difficulties: larger lesions, lesions macroscopically presenting as LST, and lesions located in the right-sided colon. A multicenter study including inexperienced endoscopists is needed to confirm the reproducibility and its effect in the training process of ESD.

## Figures and Tables

**Table 1.** Baseline Characteristics of Patients with Superficial Colorectal Neoplasms Treated by Conventional Endoscopic Submucosal Dissection (ESD) and Inner Traction-Facilitated ESD Using Rubber Band and Clip (RAC-ESD) in the Unmatched Cohorts

	Conventional ESD Group (n = 528)	RAC-ESD Group (n = 35)	*P*
Age (years)	62.8 ± 11.6	63.2 ± 10.3	.815
Gender			.889
Men (%)	293 (55.5)	19 (54.3)	
Women (%)	235 (44.5)	16 (45.7)	
Localization			.000
Rectum	305 (57.8)	5 (14.3)	
Sigmoid to descending colon	93 (17.6)	11 (31.4)	
Transverse colon to ascending colon	96 (18.2)	16 (45.7)	
Cecum	34 (6.4)	3 (8.6)	
Macroscopic type			.008
LST	241 (45.6)	24 (68.6)	
Non-LST	287 (54.4)	11 (31.4)	
Large diameter			.099
≥2cm	350 (66.3)	29 (82.9)	
<2cm	177 (33.5)	6 (17.1)	

LST, lateral spreading tumor.

**Table 2.** Baseline Characteristics of Patients with Superficial Colorectal Neoplasms Treated by Conventional Endoscopic Submucosal Dissection (ESD) and Inner Traction-Facilitated ESD Using Rubber Band and Clip (RAC-ESD) in the Matched Cohorts

	Conventional ESD Group (n = 140)	RAC-ESD Group (n = 35)	*P*
Age (years)	63.8 ± 11.0	63.2 ± 10.3	.791
Gender			.275
Men (%)	90 (64.3)	19 (54.3)	
Women (%)	50 (35.7)	16 (45.7)	
Localization			.535
Rectum	24 (17.1)	5 (14.3)	
Sigmoid to descending colon	44 (31.4)	11 (31.4)	
Transverse colon to ascending colon	66 (47.1)	16 (45.7)	
Cecum	6 (4.3)	3 (8.6)	
Macroscopic type			.935
LST	95 (67.9)	24 (68.6)	
Non-LST	45 (32.1)	11 (31.4)	
Large diameter			.701
≥2cm	122 (87.1)	29 (82.9)	
<2cm	18 (12.9)	6 (17.1)	

LST, lateral spreading tumor.

**Table 3.** Outcomes of the Patients with Superficial Colorectal Neoplasms Treated by Endoscopic Submucosal Dissection Using Rubber Band and Clip (RAC-ESD) and Conventional ESD in the Matched Cohort

	Conventional ESD Group (n = 140)	RAC-ESD Group (n = 35)	*P*
Resected area (cm^2^)	4.1 (2.8-6.9)	6.0 (2.5-9.9)	.233
Procedure time (minutes)	30.0 (16.3-55.0)	40.0 (25.0-80.0)	.031
Resection speed (cm^2^/min)	0.09 (0.04-0.16)	0.14 (0.07-0.27)	.003
En bloc resection (%)	94.3	100.0	.320
R0 resection (%)	95.0	97.1	.928
Curative resection (%)	84.3	94.3	.206
Complications (%)			.884
Perforation	2 (1.4)	0	
Delayed bleeding	1 (0.7)	0	
Pathological analysis			.104
Cancer/High grade intraepithelial neoplasia (HGIN)	71 (50.7)	11 (31.4)	
Adenoma	63 (45.0)	21 (60.0)	
Neuroendocrine tumor	6 (4.3)	3 (8.6)	

**Table 4.** Subgroup Analysis of Resection Speed

Resection Speed (cm^2^/min)	n (ESD/RAC-ESD)	Conventional ESD group (n = 140)	RAC-ESD Group (n = 35)	*P*
Localization				
Rectum	24/5	0.10 (0.07-0.16)	0.10 (0.06-0.19)	.978
Sigmoid to descending colon	44/11	0.09 (0.03-0.16)	0.13 (0.10-0.27)	.121
Transverse colon to ascending colon	66/16	0.08 (0-0.13)	0.14 (0.09-0.27)	.001
Cecum	6/3	0.13 (0.04-0.20)	0.06 (0.01-0.27)^*^	1.000
Macroscopic type				
LST	95/24	0.08 (0-0.14)	0.14 (0.07-0.27)	.001
Non-LST	45/11	0.11 (0.07-0.16)	0.11 (0.06-0.17)	.741
Large diameter				
≥2 cm	122/29	0.10 (0.04-0.17)	0.14 (0.10-0.27)	.454
<2 cm	18/6	0.05 (0-0.08)	0.05 (0.03-0.11)	.002

^*^Data are presented as median (range) due to the limited sample size.

ESD, endoscopic submucosal dissection; LST, lateral spreading tumor; RAC, rubber and clip.
